# Case Report: A case of cytomegalovirus-related hemorrhagic cystitis early after pediatric kidney transplantation

**DOI:** 10.3389/fped.2025.1665675

**Published:** 2025-11-24

**Authors:** Jianming Li, Chenglin Wu, Mengxun Wei, Peisong Chen, Xiaojun Su, Mengzhi Hong, Qian Fu, Jun Li, Longshan Liu, Changxi Wang

**Affiliations:** 1 Organ Transplant Center, First Affiliated Hospital, Sun Yat-sen University, Guangzhou, China; 2 Sun Yat-sen University, Guangzhou, China; 3 Department of Clinical Laboratory, The First Affiliated Hospital, Sun Yat-sen University, Guangzhou, China

**Keywords:** cytomegalovirus infections, hemorrhagic cystitis, pediatrics, kidney transplantation, infection

## Abstract

Cytomegalovirus (CMV) infections are commonly observed in immunocompromised patients. However, hemorrhagic cystitis (HC) is an exceptionally rare manifestation. Here, we report a pediatric kidney transplant recipient who developed CMV-related HC, presenting with acute painful macrohematuria, bladder wall thickening, and graft hydronephrosis during the early posttransplant period.

## Introduction

Hemorrhagic cystitis (HC) is defined as a diffuse inflammatory condition of the urinary bladder characterized by bleeding from the bladder mucosa (1). HC may result from both infectious and non-infectious etiologies. Infectious factors include bacteria, viruses [such as adenovirus, cytomegalovirus (CMV), and BK virus], fungi, or parasites, and non-infectious factors include chemotherapy (such as cyclophosphamide and ifosfamide), environmental toxins, and radiation (1–3). Notably, CMV is an exceptionally rare cause of HC (4). A previous report described an unusual case of late-onset CMV-related HC in a kidney transplant recipient, emphasizing its atypical clinical manifestations and the therapeutic role of intravenous ganciclovir (5). However, early-onset cases have not yet been reported. Here, we report a pediatric kidney transplant recipient who developed CMV-related hemorrhagic cystitis, presenting with acute painful macrohematuria, bladder wall thickening, and graft hydronephrosis during the early posttransplant period.

## Case

A 10-year-old boy underwent kidney transplantation from a brain-dead donor. The recipient was CMV seropositive prior to transplantation. Ureter–bladder anastomosis was performed with placement of a double-J stent, which was removed 29 days later. Induction immunosuppressive therapy consisted of basiliximab, followed by maintenance therapy with tacrolimus, mycophenolate mofetil (MMF), and prednisone. The allograft function remained stable at creatinine levels ranging from 60 to 80 µmol/L. Oral valganciclovir was administered at hospital discharge for viral prophylaxis.

**Figure 1:**
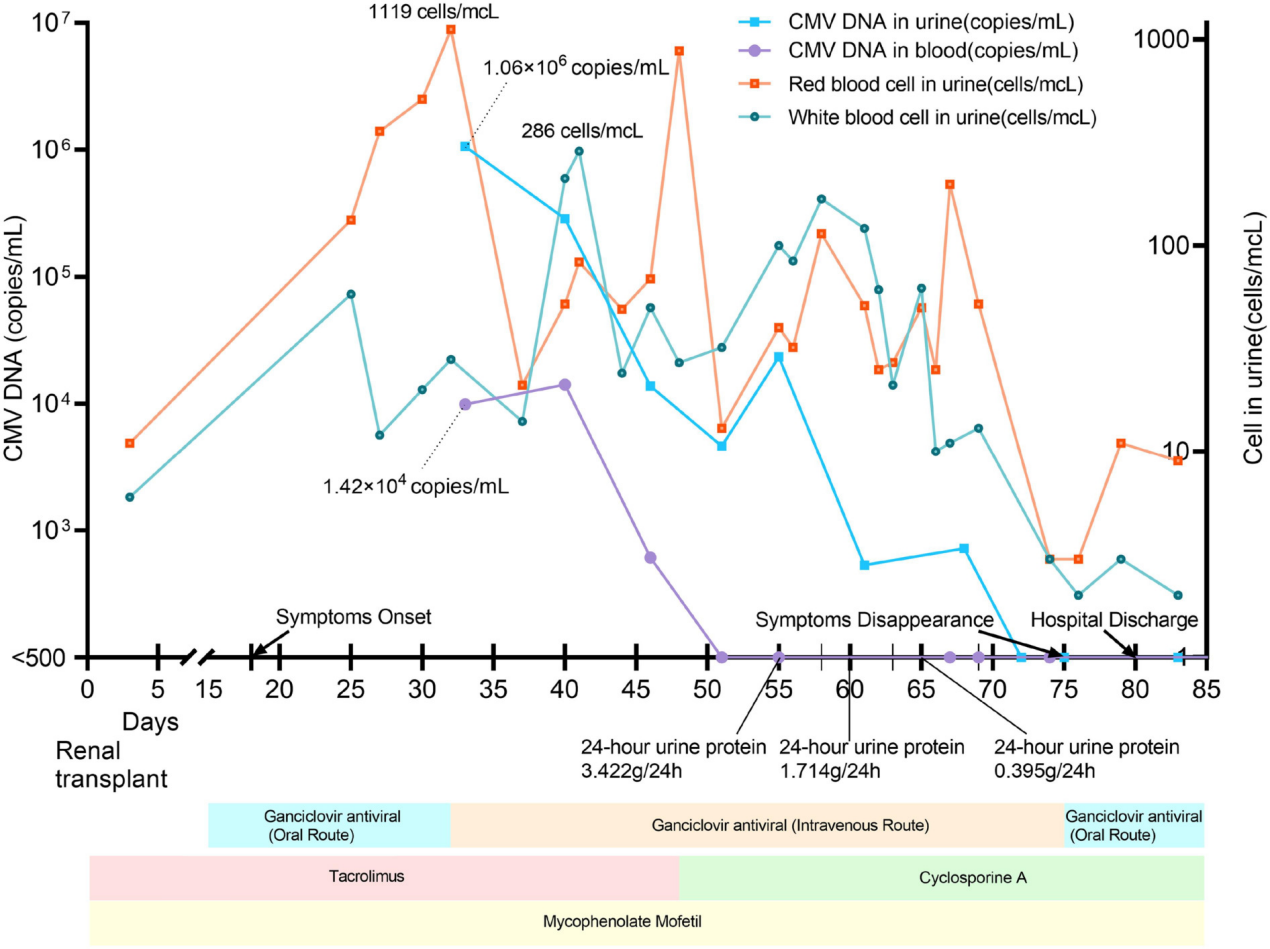
Treatments, clinical and laboratory findings according to posttransplant days. The local laboratory reference value was <500 copies/mL for both urine and plasma samples.

On postoperative day 18, he developed acute painful macrohematuria with white, purulent flocculent deposits, accompanied by pain in the lower abdomen, perineal, and scrotal regions. He also presented with noticeable overactive bladder syndrome and recurrent high fever (up to 38°C), while no other systemic abnormalities were noted. Notably, 5 days before the onset of symptoms, the tacrolimus trough concentration reached 17.7 µg/L (normal therapeutic range: 5–15 µg/L). Laboratory tests showed a maximum urine red blood cell count of 1,119 cells/µL, a maximum urine white blood cell count of 286 cells/µL, and a maximum 24 h urine protein quantification of 3.422 g/24 h. The changes in urine erythrocytes, urine protein, and urine leukocytes are shown in Figure 1. Repeated urine cultures were negative for bacterial and fungal pathogens. However, both CMV IgM and IgG were positive. The highest CMV DNA loads in blood and urine were 1.42 × 10^4^ and 1.06 × 10^6^ copies/mL, respectively (Figure 1).

**Figure 2:**
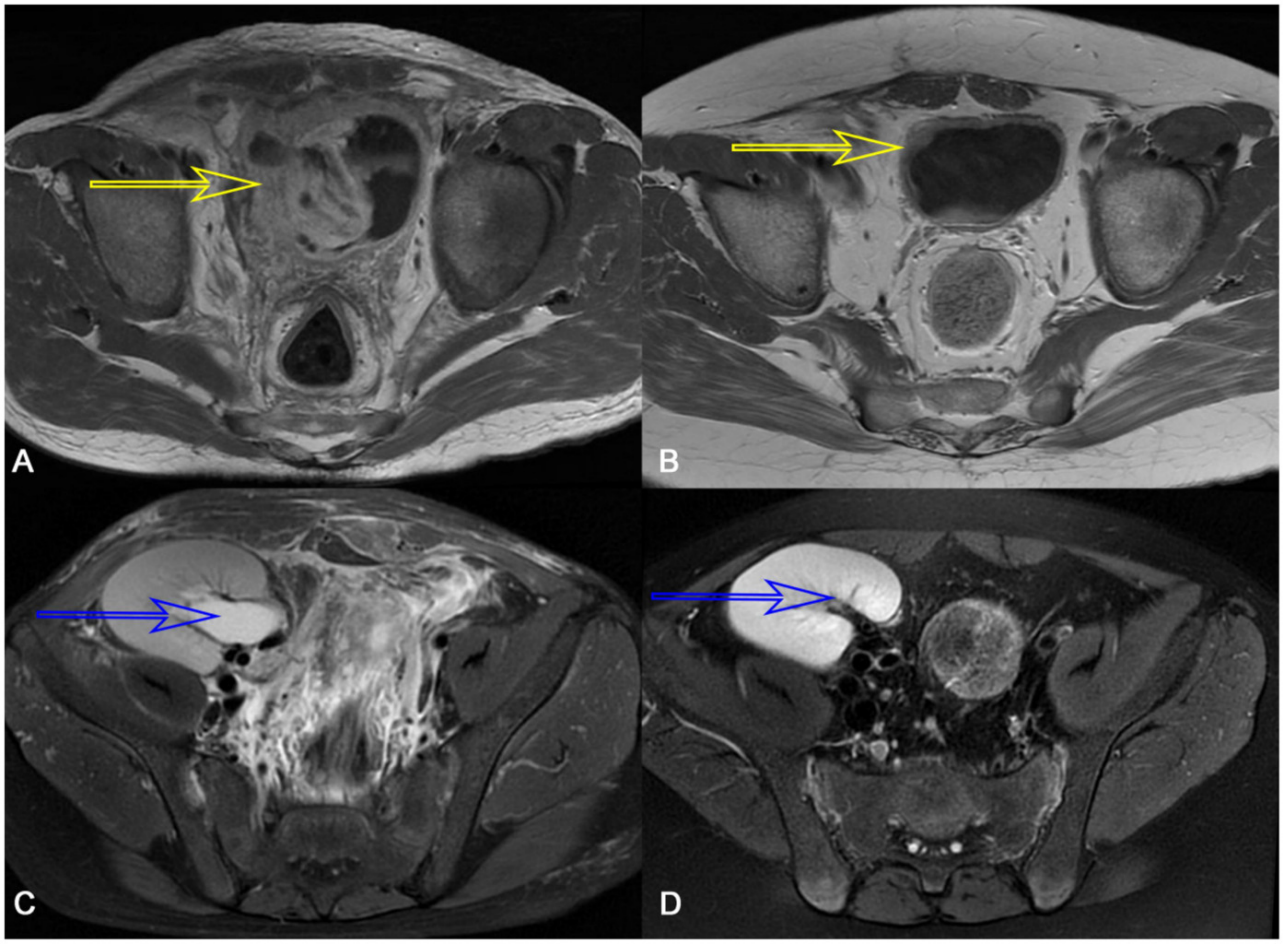
CT scan showed thickened bladder wall (yellow arrow) before treatment **(A)** and after treatment **(B)** and graft kidney hydronephrosis (blue arrow) before treatment **(C)** and after treatment **(D)**.

**Figure 3:**
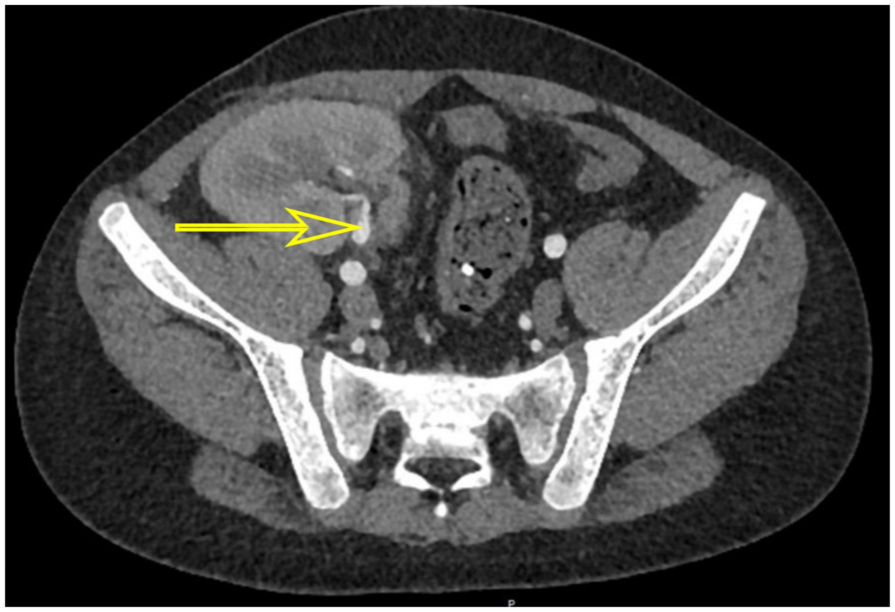
CT scan showed transplanted renal artery stenosis (yellow arrow).

On posttransplant days 39 and 60, he underwent cystoscopy, and both procedures revealed HC features of clots in the bladder. The first bladder mucosa biopsy pathology revealed a suspicious nuclear pseudoinclusion. However, immunohistochemistry staining was negative for CMV. The second bladder mucosa biopsy showed no evidence of viral inclusion bodies, as did urine pathology. Although these findings did not provide a clear diagnosis of CMV infection, his metagenomic next-generation (mNGS) sequencing of bladder tissue indicated the presence of CMV and Torque teno virus. Furthermore, polymerase chain reaction (PCR) testing of the bladder tissue revealed a peak CMV DNA load of 1.06 × 10^3^ copies/mL, indicating active viral replication. Computed tomography (CT) demonstrated bladder wall thickening and graft hydronephrosis (Figures 2, 3).

After ruling out other possible causes of hematuria and identifying CMV in bladder tissue biopsies by mNGS and PCR testing, the patient was diagnosed with CMV-related HC. At treatment initiation, intravenous ganciclovir was administered, tacrolimus was switched to cyclosporine, and prednisone was temporarily withheld to restore antiviral immune competence. Once the viral DNA load began to decline, intravenous ganciclovir was transitioned to oral administration. Due to the adjustment of the immunosuppressive protocol and antiviral drug regimen, CMV viral load declined. On posttransplant day 75, his symptoms generally disappeared. Laboratory tests showed the disappearance of urine protein, a blood creatinine level of 53 µmol/mL, and negative results for CMV detection in urine and blood. On posttransplant day 80, he was discharged from the hospital without recurrence of CMV-related HC. Follow-up examinations conducted 17 months after transplantation revealed chronic inflammatory changes of the bladder wall with marked improvement and resolution of graft hydronephrosis (Figure 2).

## Discussion

CMV-related hemorrhagic cystitis is more commonly observed in pediatric and immunocompromised patients. Viral infections represent an important cause of hemorrhagic cystitis, with CMV being one of the implicated pathogens (1). Due to the disproportion between the high prevalence of CMV-seronegative recipients and the increased proportion of CMV-seropositive donors in pediatric transplantation, this population is at a particularly elevated risk of CMV infection (6). However, in our case, the recipient was pretransplant CMV seropositive, whereas donor serology was not performed. In addition, tacrolimus overexposure may lead to an excessive immunosuppressive burden, potentially contributing to CMV-related HC. Notably, all but one of the previously reported CMV-related HC cases occurred in immunosuppressed patients (7). Regrettably, lymphocyte subset counting was not performed during the onset of HC. In addition, no other risk factors for HC (1) were identified in this patient. These findings highlight the importance of prophylactic antiviral therapy, particularly in pediatric recipients at increased risk of CMV-related HC.

PCR and mNGS tests are important diagnostic tools for CMV-related HC. Cystoscopy can help identify inflammatory changes in the bladder and pinpoint bleeding sites, while CMV infection can be confirmed through bladder biopsy specimens using culture, immunohistochemical staining, or *in situ* hybridization. However, cystoscopy and bladder mucosal biopsy are invasive tests and should be chosen carefully. Furthermore, it has been reported that CMV can only be detected in deep biopsies (8), meaning that the diagnostic yield can be limited by biopsy depth. The bladder tissue is bulging due to chronic inflammation, making it difficult to retrieve the CMV virus from the lesion in our case. Given the difficulties in diagnosing CMV-related HC, a combination of mNGS and PCR tests is necessary.

Hemorrhagic cystitis has been well described in four grades (9) and may present with serious complications. In addition to manifesting as urinary tract symptoms such as painful hematuria, CMV-related HC also produces systemic symptoms of CMV infection. In patients who have undergone kidney transplantation, CMV-related HC may lead to hydronephrosis of the graft kidney. Bladder spasm due to CMV-related HC and bladder wall thickening contributed to the hydronephrosis in our study. In addition, CMV ureteritis as a cause of ureteral obstruction may also lead to hydronephrosis (10, 11). In our case, the presence of proteinuria, which was not related to the primary disease, improved with effective CMV treatment. We consider that this may have been caused by kidney injury resulting from the CMV infection.

Timely adjustment of immune status and antiviral drug regimen was crucial for the successful treatment of CMV-related HC. In our study, we promptly switched from tacrolimus to cyclosporine and discontinued prednisone, which together reduced immunosuppressive intensity and helped control viral replication (12). Our study also confirms the effectiveness of changing tacrolimus to cyclosporine for CMV-related HC. In addition, timely intravenous administration of antiviral drugs was essential to virus elimination. Despite the improved symptoms, laboratory parameters fluctuated repeatedly in this case; this separation phenomenon suggests that early treatment is crucial to avoid the transition to chronic infection.

## Conclusions

We present a case study of a pediatric kidney transplant recipient who experienced acute painful macrohematuria, bladder wall thickening, and graft kidney hydronephrosis soon after transplantation. The detection of CMV through bladder mucosal biopsy was challenging, and combining mNGS and PCR tests can better detect microorganisms. Moreover, prompt adjustments to the immunosuppressive protocol and antiviral drug regimen resulted in improved bladder symptoms and graft kidney hydronephrosis, with no recurrence.

## Data Availability

The raw data supporting the conclusions of this article will be made available by the authors, without undue reservation.

## References

[1] Manikandan R Kumar S Dorairajan LN. Hemorrhagic cystitis: a challenge to the urologist. Indian J Urol. (2010) 26(2):159–66. 10.4103/0970-1591.65380 20877590 PMC2938536

[2] Khan AM Ajmal Z Tuz Zahra F Ramani A Zackon I. Hemorrhagic cystitis secondary to adenovirus and BK virus infection in a diffuse large B-cell lymphoma patient with recent CAR T-cell therapy. Case Rep Hematol. (2020) 2020:6621967. 10.1155/2020/6621967 33294236 PMC7717988

[3] Haldar S Dru C Bhowmick NA. Mechanisms of hemorrhagic cystitis. Am J Clin Exp Urol. (2014) 2(3):199–208. 25374922 PMC4219308

[4] Padayachee WPR Sadhwani S Doherty SW Mukendi AM Van den Berg E Botha AR. Haemorrhagic cystitis due to cytomegalovirus in a patient with AIDS. Afr J Urol. (2020) 26:30. 10.1186/s12301-020-00039-4

[5] Ersan S Yorukoglu K Sert M Atila K Celik A Gulcu A Unusual case of severe late-onset cytomegalovirus-induced hemorrhagic cystitis and ureteritis in a renal transplant patient. Ren Fail. (2012) 34(2):247–50. 10.3109/0886022X.2011.647209 22251223

[6] Höcker B Zencke S Krupka K Fichtner A Pape L Dello Strologo L Cytomegalovirus infection in pediatric renal transplantation and the impact of chemoprophylaxis with (val-)ganciclovir. Transplantation. (2016) 100(4):862–70. 10.1097/TP.0000000000000888 26736017

[7] Taktak A Acar B Gür G Tiryaki T Karakuş E Çaycı FŞ Cytomegalovirus-related hemorrhagic cystitis in an immunocompetent child. Ren Fail. (2014) 36(7):1148–50. 10.3109/0886022X.2014.926757 24932852

[8] Whitaker JA Jacob JT Little JV DiazGranados CA. Cytomegalovirus cystitis with bladder wall dehiscence in a patient with AIDS. Aids. (2008) 22(6):795–6. 10.1097/QAD.0b013e3282f56103 18356616

[9] Cipe FE Soygür T Doğu F Erdoğan O Bozdoğan G Ikincioğullari A. Late onset hemorrhagic cystitis in a hematopoietic stem cell recipient: treatment with intravesical hyaluronic acid. Pediatr Transplant. (2010) 14(6):E79–82. 10.1111/j.1399-3046.2009.01169.x 19344339

[10] Ardalan M. Rare presentations of cytomegalovirus infection in renal allograft recipients. Nephrourol Mon. (2012) 4(2):431–6. 10.5812/numonthly.1844 23573461 PMC3614274

[11] Rico JE Cardona X Rodelo J Reino A Arias LF Arbeláez M. Ureterostomy cytomegalovirus infection presenting as stoma ulceration in a kidney allograft receptor: a case report. Actas Urol Esp. (2008) 32(6):649–52. 10.1016/S0210-4806(08)73903-2 18655351

[12] Kizilbash SJ Rheault MN Bangdiwala A Matas A Chinnakotla S Chavers BM. Infection rates in tacrolimus versus cyclosporine-treated pediatric kidney transplant recipients on a rapid discontinuation of prednisone protocol: 1-year analysis. Pediatr Transplant. (2017) 21(4):e12919. 10.1111/petr.12919 PMC5423828 28371243

